# Brazilian Vaccinia Viruses and Their Origins

**DOI:** 10.3201/eid1307.061404

**Published:** 2007-07

**Authors:** Giliane S. Trindade, Ginny L. Emerson, Darin S. Carroll, Erna G. Kroon, Inger K. Damon

**Affiliations:** * Centers for Disease Control and Prevention, Atlanta, Georgia, USA; † Federal University of Minas Gerais, Belo Horizonte, Minas Gerais, Brazil; 1 These authors contributed equally to this work.

**Keywords:** Vaccinia virus, genetic diversity, Brazil, perspective

## Abstract

Although the World Health Organization (WHO) declared global smallpox eradicated in 1980, concerns over emergent poxvirus infections have increased. Most poxvirus infections are zoonotic; exploring their genetic diversity will illuminate the genetic and evolutionary aspects of poxvirus infections, ecology, and epidemiology. In recent decades, several strains of the orthopoxvirus vaccinia virus (VACV) have been isolated throughout Brazil, including many genetically distinct isolates within the same outbreak. To further investigate the diversity and origins of these viruses, we analyzed molecular data from 8 Brazilian VACV isolates and compared several genes involved in virus structure and pathogenicity. Genetic variation among isolates suggests that ancestral Brazilian VACVs existed before the beginning of the WHO smallpox eradication vaccination campaigns and that these viruses continue to circulate.

## Abstract 2

Genetic diversity enables this virus to persist in Brazil and other parts of the world.

In 1980, the World Health Organization (WHO), after a massive vaccination program, announced the eradication of smallpox, the contagious and deadly disease caused by *Variola virus* (VARV). This program used live vaccinia virus (VACV), a virus from the same genus, orthopoxvirus, which shares a high degree of immunologic cross-reactivity with VARV. Recent reports of cowpox virus (CPXV) infections in Europe, monkeypox virus (MPXV) outbreaks in Africa and the United States, and the surprising emergence of VACV in Brazil highlight the need for continued research into the ecology, epidemiology, origin, and evolution of these viruses (*1*).

The known history of VACV species imported to Brazil dates back to 1804, when human vaccine arrived at a port in Bahia State on the arms of slaves returning to Brazil from Lisbon, Portugal (*2*) (Figure 1). From Bahia State the slaves were sent south to Rio de Janeiro State, possibly passing the vaccine to local people as they made their way through the region. Indeed, from 1804 to 1887, the Brazilian population (including slaves and other people living in the countryside) was vaccinated in this manner, arm to arm. In some cities, vaccination was obligatory, beginning in 1832. In 1887, the first animal vaccine produced in calves was imported in flasks to the vaccine institute in Rio de Janeiro (now Oswaldo Cruz Institute) from the Chambon Institute in Paris, France (*2*). The vaccine was then distributed to other states across Brazil, including Minas Gerais, Espirito Santo, São Paulo, Mato Grosso, Rio Grande do Sul, and Pernambuco (*2*). During this time, between 1887 and 1895, vaccine institutes were established in these states (*2*).

**Figure 1:**
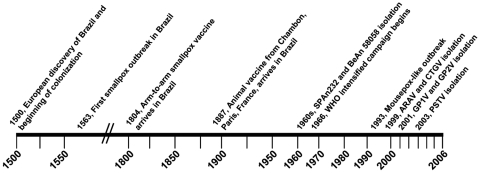
Timeline of events regarding the introduction and circulation of orthopoxviruses in Brazil (*2*,*3*). Double slashes indicate a gap in the timeline.

Published accounts of Brazilian vaccinia-like viruses isolated from sentinel mice and recent outbreaks on dairy farms affecting cattle and their handlers suggest the once-emergent disease has now become endemic (*4*–*13*). We combined historical information and published data to develop insights into the possible origin(s) of the Brazilian VACV (BRZ-VACV) now established in Brazil. Our goal was to determine whether BRZ-VACV represents an escaped vaccine strain, an autochthonous orthopoxvirus, or both.

## Methods

We examined the sequence diversity of 8 geographically and temporally variable BRZ-VACV isolates. We compared molecular sequence data from 3 genes and a variable region of the poxvirus genome (Table 1) among BRZ-VACV isolates, available vaccine strains related to those used during the eradication campaign in Brazil, and other VACVs isolated from domestic animals (including endemic buffalopox virus in India [*15**–**17*] and horsepox virus [HSPV] from Asia [*18*]).

**Table 1:** Single gene sequences of *Vaccinia* viruses included in this study

Strain, isolate (abbreviation)	Gene and GenBank accession no.*			
	A56R	B19R	E3L	Rpo132-ATI-p4c-A27L
BeAn 58058 virus (BAV)	DQ206442	AF261890	DQ194388	NA
SpAn232 virus (SAV)	AF261890	DQ194384	DQ194387	NA
Belo Horizonte (VBH)	DQ206435	DQ194383	DQ194390	AF501620
Guarani P1 virus (GP1V)	DQ206436	DQ194380	DQ194385	DQ363383
Guarani P2 virus (GP2V)	DQ206437	DQ194381	DQ194386	NA
Araçatuba virus (ARAV)	AY523994	DQ194382	DQ194389	NA
Passatempo virus (PSTV)	DQ070848	DQ530239	DQ530240	NA
Cantagalo virus (CTGV)	AF229247	AY500815	AY771338	NA
Malbran virus (VACV-Malbran)	AY146624	NA	NA	NA
Vaccinia virus-Oswaldo Cruz Institute (VACV-IOC)	AF229248	AY500816	DQ070236	NA
BFL-3906	AF375077	NA	NA	NA
BFL-81	AF375078	NA	NA	NA
Wyeth	Z99051	Not included	NA	NA

*Reference: Vaccinia Copenhagen. A56R, viral hemagglutinin; B19R, soluble alpha/beta interferon [IFN] receptor; E3L, dsRNA-binding protein; rpo132-ATI-p4c-A27L, a region that codes for the major protein of the A-type inclusion body (*14*); NA, not available.

Sequences were collected from GenBank (Tables 1 and 2). Some isolates had limited sequence data for the 4 genes examined. Although all 4 genes are not available from Lister (B19R gene is not present in the Lister genome), the Lister isolate was included because of its importance in the history of vaccination in Brazil (*3*,*6*). Sequences from 3 genes were manually aligned and trimmed to include only the regions available for all isolates (VACV-COP A56R 161210–162095, E3L 51465–50929, B19R 178242–179173). Nucleotide identities were calculated by using BioEdit (www.mbio.ncsu.edu/bioedit/bioedit.html) (Technical Appendix**).** PAUP* version 4 (Sinauer Associates; Sunderland, MA, USA) and MrBayes 3.1 (http://mrbayes.csit.fsu.edu) were used to analyze a matrix of the 3 concatenated genes (*19**,**20*). Parsimony analysis was performed on 1,000 random addition replicates. Appropriate models of sequence evolution were determined by Modeltest (*21*) with the Akaike information criterion (*22**,**23*). The identified model was then applied to maximum likelihood and Bayesian analyses. In MrBayes, the Markov chain Monte Carlo searches explored 4 chains for 1 million generations, sampling every 100 generations. Sample points during the first 50,000 generations were discarded as burn-in, before which the chain reached stationarity (fixed condition). To account for any considerable rate variation between genes, a second Bayesian analysis was run wherein each gene was partitioned and allowed to vary independently (MrBayes command: prset ratepr = variable). DNA sequence data for the fourth coding region, rpo132-ATI-p4c-A27 (A-type inclusion body gene), were not included in the phylogenetic analysis; however, published digestion profiles from relevant strains were compared.

**Table 2:** Complete virus genome sequences of orthopoxviruses

Species	Strain, isolate (abbreviation)	**GenBank accession no.**
Vaccinia virus	3737 (VACV-3737)	DQ377945
	Acambis 3000 (VACV-Acambis 3000)	AY603355
	Acambis 2000 (VACV-Acambis 2000)	AY313847
	Acambis 3 (VACV-Acambis 3)	AY313848
	Vaccinia DUKE (VACV-DUKE)	DQ439815
	Lister (VACV-LIS)	AY678276
	Tian Tan (VACV-TianTan)	AY678275
	Western Reserve (VACV-WR)	AY243312
	Modified Vaccinia Ankara (VACV-MVA)	U94848
	Copenhagen (VACV-COP)	M35027
Horsepox virus	HPXV MNR-76 (HPXV)	DQ792504
Rabbitpox virus	Utrecht (RPXV-UTR)	AY484669
Cowpox virus	Brighton Red (CPXV-BR)	AF482758
	GRI-90 (CPXV-GRI)	X94355
Variola virus	Bangladesh-1975 (VARV-BSH 75)	L22579
	Garcia-1966 (VARV-GAR)	X76266
	India-1967 (VARV-IND)	X69198
Monkeypox virus	Congo_2003_ (MPX-RCG 2003)	DQ011154
	Liberia_1970_184 (MPXV-LIB 1970)	DQ011156
	USA_2003_039 (MPXV-USA 2003 039)	DQ011157
	WRAIR7-61 (MPXV-61 WR)	AY603973
	Zaire_1979-005 (MPXV-ZAI 1979)	DQ011155
	Zaire-96-I-16 (MPXV-ZAI 1996)	AF380138
Ectromelia virus	Moscow (ECTV-MOS)	AF012825
Camelpox virus	CMS (CMLV-CMS)	AY009089
	M-96 (CMLV-M96)	AF438165

## Results

**Figure 2:**
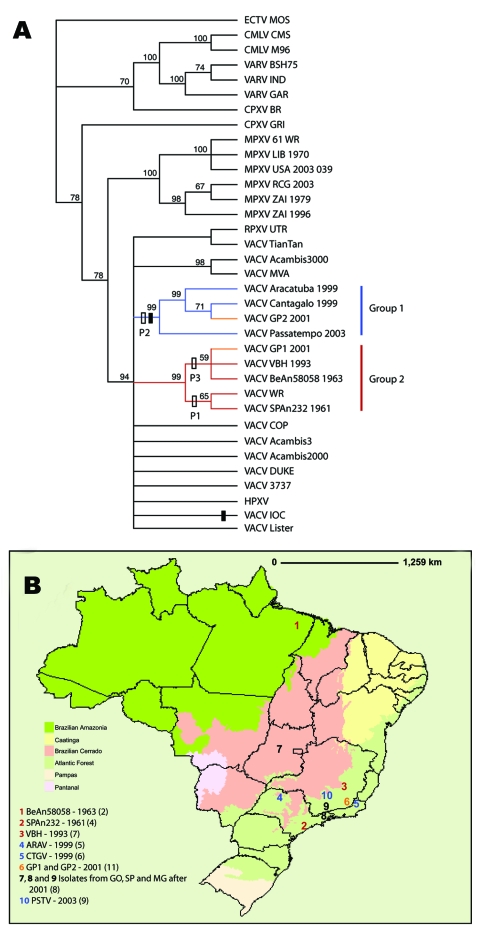
A) Strict consensus of the 6 most parsimonious trees derived from *Orthopoxvirus* species on the basis of B19R, E3L, and A56R sequences and rooted with ectromelia virus from Moscow (ECTV-MOS). Bootstrap values based on 100 bootstrap replicates of 10 random addition replicates each are shown at each node where the value was >50%. Black rectangles represent the 18-nt deletion shared by group 1 Brazilian vaccinia virus (BRZ-VACV) and VACV-Institute Oswaldo Cruz (IOC) and where it maps on the tree. Open rectangles represent the 3 digestion profiles (P1, P2, and P3) of the rpo132-ATI-p4c-A27L region assigned at each appropriate node. Blue, branches of group 1; red, branches of group 2; orange, isolation of VACV-Guarani P1 (GP1) and VACV-GP2 at adjacent farms in the same area. Colors correspond to information presented in panel B. B) Map of Brazil showing states and ecological biomes. Collection sites are represented by numbers that refer to Brazilian isolates and their respective years of isolation. Map used with permission from Instituto Brasileiro de Geografia e Estatistica. GO, Goiás; SP, São Paulo; MG, Minas Gerais. See Table 2 for other definitions.

The resultant phylogenetic tree (Figure 2A) depicts several highly supported clusters including monophyletic VACV, MPXV, VARV, and camelpox virus (CMLV) assemblages. VARV and CMLV isolates are depicted as sister taxa with high support. Brazilian isolates fall into 2 well-supported monophyletic groups. Group 1 comprises Araçatuba virus (ARAV), Cantagalo virus (CTGV), Guarani P2 virus (GP2V), and Passatempo virus (PSTV); group 2 comprises GP1V, Belo Horizonte virus (VBH), BeAn 58058 virus (BAV), SPAn232 virus (SAV), and VACV-Western Reserve (WR). Results of all analyses—parsimony, maximum likelihood, and Bayesian analysis (data not shown)—all generated 2 distinct Brazilian clades and indicated a close relationship between group 2 and VACV-WR. Neither group of BRZ-VACV was directly linked with VACV-Oswaldo Cruz Institute (IOC) or VACV-Lister in any analysis. Both BRZ-VACV groups are represented by 4 isolates each. VACV-Acambis3000 and VACV-modified vaccinia Ankara (MVA) group together with high support (98, Figure 2A), which reflects their derivation from vaccinia isolate Ankara (both are virus isolates of MVA). Isolates with known origins from Dryvax (Wyeth Laboratories, Marietta, PA, USA) (VACV-3737, -Acambis3, -Acambis2000, -DUKE) did not group together consistently. This could be a result of numerous passages, the history of Dryvax as a nonclonal vaccine strain, or both.

Genetic comparisons of the analyzed VACV strains can be seen in the Technical Appendix. For BRZ-VACV at all 3 loci, the highest identity values occurred within each phylogenetic group. The E3L sequence of PSTV is the only exception. The PSTV E3L sequence is identical to those of GP1 and WR and showed the next highest identity level with the VBH sequence. This finding may indicate recombination in this portion of the genome between isolates from these 2 groups. Additional genomic data and analysis are needed.

**Figure 3:**
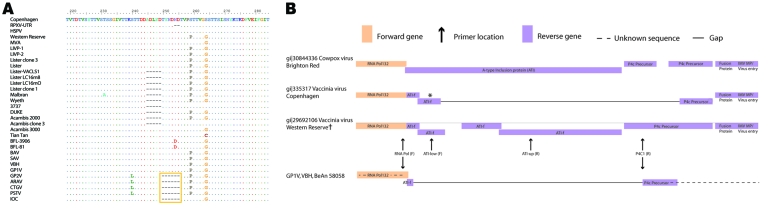
A) Partial amino acid alignment of A56R vaccinia virus Copenhagen (VACV-COP). Sequences were retrieved from GenBank and aligned using the BioEdit program (www.mbio.ncsu.edu/bioedit/bioedit.html). VACV-COP (M35027, 161908-161957) is used as the reference sequence; International Union of Pure and Applied Chemistry symbols, amino acids; dots, residues identical to the reference sequence; dashes, gaps created by the alignment; gold box surrounds the 18-nt deletion once proposed as a Brazilian (BRZ)-VACV molecular identifier. RPXV-UTR, rabbitpox virus-Utrecht; HSPV, horsepox virus; MVA, modified vaccinia Ankara; LIVP, Liverpool; BFL, buffalopox; BAV, BeAn 58058 virus; SAV, SpAn232 virus; VBH, Belo Horizonte virus; GP, Guarani virus; ARAV, Araçatuba virus; CTGV, Cantagalo virus; PSTV, Passatempo virus; IOC, Institute Oswaldo Cruz. B) Depiction of the rpo132-ATI-p4c-A27 region in Cowpox virus Brighton Red (CPXV-BR) and VACV strains. Positions of primers RNApol, ATI-low, ATI-up, and P4c1 are illustrated. These primers have been used for the molecular diagnosis of Brazilian (BRZ)-VACV (*5*,*26*). The 300-bp fragment amplified by primers RNApol and P4c1 from GP1V and VBH was sequenced by Trindade et al. (*9*,*13*). Sequence outside this region is unknown. ATI-f is used to designate coding regions that resemble fragments of the wild-type (CPXV-BR) ATI gene. The gap in VACV-COP lies within the A26L gene. *This gene is often referred to as ATI due to its original description. However, the coding region is actually a fusion of the C-terminus of ATI and the N-terminus of P4c where the sequence in between has been deleted. The gene is currently identified by the Poxviridae Bioinformatics Resource (http://athena.bioc.uvic.ca/database.php?db=poxviridae) as belonging to the P4c precursor family. †Western Reserve is presumed similar in structure to SpAn232 virus, GP2V, ARAV, and PSTV due to similar restriction fragment length polymorphism results (*6*,*7*,*11*,*13*).

The sequence diversity of the orthopoxvirus hemagglutinin (HA) gene has made it a potential marker for molecular diagnostics and phylogenetics (*24*). In particular, an 18-nt deletion within the gene has been proposed as an identifier of BRZ-VACV strains (*8**,**11**,**25*). A recent article describes 2 approaches for identifying BRZ-VACV–like isolates (*25*), both of which rely on presence of the 18-nt deletion. Figure 3 shows that while some Brazilian strains (ARAV, CTGV, GP2V, PSTV) share this deletion, others (SAV, BAV, GP1V and VBH) do not and would not be detected by these methods. Within the variably deleted region (Figure 3, amino acids 245–255), VACV isolates can be grouped into 4 types; Brazilian isolates fall into 2 of these. Rabbitpox virus from Utrecht has a unique deletion of only 2 residues (254, 255); VACV-3737 and VACV-Malbran share a 5–amino acid deletion (245–249). The VACV-3737 isolate was “plaque-purified from a vaccinia lesion following vaccination with Dryvax” (GenBank,accession no. DQ377945); VACV Malbran was used in vaccination programs in Argentina between 1937 and 1970 (*27*). GP1V and VBH share the same amino acid sequence (ADLYDTYNDND; 245–255), identical to that of VACV-WR, VACV-Lister, and several other strains. The remaining isolates from Brazil share the familiar deletion of 6 amino acids (250–255) described previously. This deletion is also present in the VACV-IOC vaccine isolate (absent in Lister and VACV-WR). The variation in this region demonstrates that the deletion itself is not representative of all Brazilian isolates and therefore is not useful as an identifier of Brazilian VACV.

This same deletion has been recognized as a possible shared derived trait between VAVC-IOC and BRZ-VACV. Although this scenario is indeed possible for the CTGV-like BRZ-VACV of group 1, those isolates of group 2 are unlikely to be derived from the IOC vaccine strain because this strain does not carry the insertion. Furthermore, another variable gap occurs in this region of HA among isolates derived from VACV-Lister and Dryvax (New York City Board of Health [NYCBH]). Some clones present the deletion; others do not. The sequence can be found directly adjacent upstream of the variable deletion of the group 1 Brazilian isolates (Figure 3). Perhaps this region is nonessential and able to easily withstand such variation without detrimental effects on the utility of the HA protein or the fitness of the virus. Regardless, this deletion clearly cannot be relied upon to identify isolates directly related to Lister or NYCBH strains. A single shared deletion is only 1 character toward determining phylogenetic relatedness among strains. If smallpox vaccines are not clones but rather pools of virions that vary at the molecular level, assuming relatedness based on a single molecular variation is even more difficult. Our phylogenetic analyses of 302 informative characters, including variation across the HA gene, does not indicate a close relationship between VACV-IOC and BRZ-VACV. The consensus tree (Figure 2A) depicts the IOC vaccine strain in an unresolved basal polytomy within the vaccinia cluster and is thus inconclusive as to the relatedness of VACV-IOC and BRZ-VACV.

Finally, published data indicate a large amount of variability in the region that includes the A-type inclusion body gene of the BRZ-VACV isolates (*5**,**9**,**11**–**13*). Figure 3B illustrates the arrangement of open reading frames in this region (rpo132-ATI-p4c-A27L) for CPXV Brighton Red, VACV Copenhagen, VACV-WR, and BRZ-VACV. The formation of A-type inclusion bodies is restricted to cells infected with CPXV, ectromelia virus, and raccoonpox virus. The C-terminus of the gene encoding the ATI protein is highly conserved among orthopoxviruses because it overlaps with the C-terminus of the RNA polymerase (132) coding region (*9**,**13*). However, the region between this conserved sequence and the N-terminus of the P4c precursor shows considerable variation among orthopoxvirus strains (*26*). Deletions and nucleotide changes generate variable coding regions and distinguishable digest patterns in this portion of the genome. Three profiles of this region have been discerned among BRZ-VACV; 2 are differentiated by distinct digestion patterns (*6**,**7**,**11**,**13*). The third state is the near complete absence of the A26L gene except for the last 112 nt (*9**,**13*). According to da Fonseca et al., SAV, a Brazilian VACV isolate, shows an A26L digestion profile identical to that of the VACV-WR strain (profile 1) (*6*). ARAV, PSTV, and GP2V show a digestion profile compatible with but not identical to that of the SAV, VACV-WR, and Lister strains (profile 2) (*7**,**11**,**13*). In BAV, VBH, and GP1V, a major portion of the A26L gene is missing (profile 3) (*5**,**9**,**13*). The open reading frame is small and is probably not expressed, although this has not been verified by Northern or Western blot. Damaso and colleagues demonstrated that A26L is present in CTGV; however, the digestion profile has not been established. Western blot analysis detected a 94-kDa protein typical of VACV-WR and VACV-IOC (*8*). These 3 profiles correspond to 3 clades recovered in the phylogenetic tree (Figure 2A). These findings constitute a major distinction between CTGV (also SAV, ARAV, PSTV, and GP2V, which exhibit profiles 1 and 2) and the other Brazilian isolates BAV, VBH, and GP1V (profile 3). Furthermore, the unusual deletion presented by BAV, VBH, and GP1V is remarkably different from the corresponding regions of VACV-IOC, -WR, and -Lister, strains implicated in the release of VACV in Brazil.

## Discussion

Although eradication had been achieved in Europe by 1953 and in Central and North America by 1951, Brazil did not conduct a nationwide vaccination campaign during the 1950s. However, Brazilian people in cities and towns were vaccinated when outbreaks were reported by local authorities (*2**,**3*). Not until 1962 did Brazil, working with the Pan American Health Organization, launch a national campaign against smallpox (*3*). The intensified global WHO program prompted a renewed national program in Brazil in 1966. Fenner et al. (*3*) present a sample questionnaire that the smallpox eradication unit circulated in 1967 to all vaccine producers in countries accessible to WHO. In Brazil, this included 4 laboratories in different regions: the IOC, the Butantan Institute in São Paulo, the Institute for Biological Research in Porto Alegre, and an institute in the town of Recife, Pernambuco State (*3*). Records identify the seed strains used at these institutes to be “Paris,” Lister, and “Lederle” (*28*). “Paris” appears to be a reference to the original animal vaccine imported from the Chambon Institute in 1887. The Institute for Biological Research in Porto Alegre received from London the Lister strain, which was originally developed at the Lister Institute, England. Recife and Butantan both indicated use of a strain obtained from Wyeth’s Lederle Laboratory in the United States. The NYCBH strain was the source of Lederle and forerunner of Dryvax, the live vaccine maintained in the United States since the 1970s, and the research vaccine strain VACV-WR (*3*). These records confirm information that “Paris,” Lister, and NYCBH were used in Brazil between 1968 and 1971 (*2*,*3*). It has been suggested that VACV-WR was used as a vaccine in Brazil as well; however, such use is now thought to be unlikely (*6**,**7*). The strain was derived by serial passage in mice infected intracranially and selected for neural virulence to mimic encephalitis, a rare side effect of the vaccine in humans (*29**,**30*). When available vaccine supplies dropped to critical levels in 1970, Brazil requested and received reserve vaccine from Argentina at least once (*3*). The Malbran strain was used in vaccination programs in Argentina until 1970, after which the Lister strain was adopted following WHO recommendations (*2**,**3**,**27*). Vaccine production at the Butantan Institute converted to the Lister strain late in 1970 (*3*), and Butantan became the official distributor for Brazil. Therefore, at least 4 strains of vaccine might have been distributed within Brazil during the WHO eradication campaign.

### Discovery of Brazilian VACV Isolates

Since the 1960s, orthopoxviruses have been repeatedly isolated in Brazil and identified as VACV by classical immunologic, virologic, and molecular methods (*4**–**13**,**26*). In 1963, BAV was isolated from the blood of a rice rat (*Oryzomys* sp.*)* captured near the edge of a deforested area bordered by Amazon rain forest (Figure 2B) (*4**,**5*). This virus is among the first orthopoxviruses naturally isolated from a wild rodent in Brazil (*4**,**5*). In the 1960s and 1970s, the Brazilian government and the Institute Adolfo Lutz conducted surveillance of arboviral activity in forested areas around the city of São Paulo, in the southeastern region of the country. During that investigation, a poxvirus was repeatedly isolated from sentinel mice and called Cotia virus (*31*). A sample of Cotia, SAV, was sent to the virus laboratory in Minas Gerais State. The specimen was plaque purified in duplicate, and a vaccinialike virus was isolated (*6*). Samples of Cotia have been studied elsewhere, and independent characterizations of the virus have been contradictory (*32**–**34*). These contradictory findings suggest that the material provided in the original sample(s) contained >1 virus, leading to conflicting reports of behavior and serologic relationships. We continue to refer to this sample as SPAn232 with reference to de Souza Lopes et al. (*31*) and da Fonseca et al. (*6*) to distinguish it from other isolates described previously.

In 1998, VBH was isolated from frozen clinical samples collected from mice during a mousepox-like outbreak in the animal facility of the Biological Institute of the University of Minas Gerais State in 1993 (Figure 2B) (*9*). Exanthematous outbreaks affecting dairy cattle and their handlers were reported in Brazil in 1999. Two new VACV strains, ARAV and CTGV, were isolated from sick cows in distinct geographic locations of the southeast region of the country (Figure 2C) (*7**,**8*). Since then, an increasing number of similar zoonotic outbreaks have been reported in different countryside areas of Brazil, particularly the southeast and southwest (Figure 2A, B) (*10**,**12*). In 2001, GP1V and GP2V were isolated from infected cows from adjacent farms near the area where CTGV had been found. Despite their coincident locale, the 2 new isolates constitute distinctly different VACV isolates (Figure 2A, C) (*13*). In 2003, PSTV was also isolated from cows in Minas Gerais State during a subsequent bovine vaccinia outbreak (Figure 2C; Table 3 [*11*]). Isolates from group 1, similar to CTGV, have been collected more often than those from group 2 (*10**,**25*), which could reflect greater fitness, prevalence, or virulence associated with group 1; however, any explanation is speculative. The difference might eventually be attributed to an as-yet unidentified sampling bias.

**Table 3:** Brazilian *Vaccinia* viruses and vaccine strains used in Brazil*

Virus	Year of isolation	Source	Place of isolation and biome	Reference
VACV-LIS	1870	Prussian soldier	Vaccine Institute, Cologne, Germany	(*3*)
VACV-WR	1876	NYCBH strain	New York City Department of Health Laboratory	(*3*)
SAV†	1961	Rodent, sentinel mice	São Paulo State, Atlantic tropical rainforest, Brazil	(*6*)
BAV	1963	Rodent, *Oryzomys sp.*	Para State, Amazon tropical rainforest, Brazil	(*4*,*5*)
VBH	1998	Rodent, BALB-c mice	Minas Gerais State, Cerrado woodland/savanna, Brazil	(*9*)
ARAV	1999	Cow	São Paulo State, Cerrado woodland/savanna Brazil	(*7*)
CTGV	1999	Cow	Rio de Janeiro State, Atlantic tropical rainforest, Brazil	(*8*)
GP1V	2001	Cow	Minas Gerais State, Atlantic tropical rainforest, Brazil	(*13*)
GP2V	2001	Cow	Minas Gerais State, Atlantic tropical rainforest	(*13*)
PSTV	2003	Cow	Minas Gerais State, Atlantic tropical rainforest, Brazil	(*11*)
VACV-IOC	ND†	Probable Paris strain	ND	(*3*,*8*)

* VACV, vaccinia virus; LIS, Lister; WR, Western Reserve; NYCBH, New York City Board of Health; SAV, SpAn232 virus; BAV, BeAn 58058 virus; VBH, Belo Horizonte virus; ARAV**,** Araçatuba virus; CTGV, Cantagalo virus; GP, Guarani virus; PSTV, Passatempo virus; IOC, Oswaldo Cruz Institute; ND, not determined. †This virus was studied by the viral laboratory in Minas Gerais State in 1979 and cloned in Vero cells during the 1990s (*4*). It was originally collected and referred to as Cotia virus, which was repeatedly isolated throughout the 1960s and 1970s by the Adolfo Lutz Institute in Brazil (*33*).

Isolates have been obtained from 3 of 6 Brazilian biome types (Figure 2B). Most have been collected in the Atlantic Forest region, where much of the forest has been cleared for dairy farms and coffee and sugar cane plantations (*35*). Reported human cases are typically in dairy workers, but SAV was isolated from a sentinel mouse placed within the Cotia Forest, São Paulo State (*6*), and BAV was obtained from a wild-caught rodent (*Oryzomys*) in northern Brazil. These occurrences, combined with the genetically similar isolates from the southern portion of the country (2,500 km away), indicate the potential circulation of viral strains throughout Brazil and perhaps other regions of South America. Moreover, some of these genetically divergent strains have a sympatric distribution, as illustrated by the isolation of GP1V (group 2) and GP2V (group 1) from adjacent farms (*13*). Sequencing of the more rapidly evolving inverted terminal repeats of the genome would help clarify the relationship between BRZ-VACV and its hosts. The near-terminal regions of the genome contain most genes involved in host interaction (*14*) and may illuminate ways in which the host may have shaped the virus evolution. No data are available regarding host variation, epidemiology, or evidence of clinical manifestations that can distinguish between the 2 genetic BRZ-VACV groups depicted in the phylogenetic analyses.

### Potential Origins of Brazilian VACV

The possibility that the BRZ-VACV isolates originated from the spread of a smallpox vaccine strain, particularly VACV-IOC and VACV-Lister, has been proposed (*4*,*6**,**8*). A deletion in HA supports this hypothesis; but the larger portion of evidence, the phylogenetic analysis, does not. Available data offer no solid support for these proposed sources as the origin of BRZ-VACV. The VACV-IOC vaccine should be further examined to determine whether it contains virions without the 18-nt HA deletion. Historical records raise the possibility that strains currently circulating in Brazil were established from the original (first known) introduction of VACV to the region by the slave trade in the early part of the 19th century, by the introduction of the animal-cultivated vaccine in the latter part of the 19th century, or subsequently by the wave of VACV introduction brought through the smallpox eradication program in the mid 20th century. If BRZ-VACV was introduced from the Old World, multiple introductions from essentially the same source region (Europe) over ≈167 years (from at least 1804 to 1971, when vaccination in Brazil ceased) (*2**,**3**,**36*) could be responsible for the observed diversity of BRZ-VACV. Because the source of the naturally occurring isolates of Brazil may well be the same as that of the vaccine strains, the history of this viral species will be difficult to tease apart without direct sampling of naturally occurring isolates from Europe, particularly England, Germany, and France, where vaccine strains used worldwide are thought to have originated.

Vaccine escape has been hypothesized to account for other VACV isolated from domestic animals, including endemic buffalopox in India and HSPV (MNR-76) in Mongolia (*15**–**18*). A limited comparison between the Brazilian sequences and HSPV did not produce a monophyletic group, which makes it unlikely that they are derived from a very recent common ancestor such as one of the common vaccine strains developed in the past century. HSPV currently has the only complete genome available for comparison from a naturally occurring isolate. As the initial phylogenetic analysis of this sample suggests, we may find potential sources, such as group 1 of the BRZ-VACV, for vaccine strains circulating in various parts of the globe. Better sampling of naturally occurring VACV will be essential for determining the existence of indigenous VACV in Brazil.

The existence of 2 distinct groups of BRZ-VACV is clear. Their origins and how they are related remain undetermined. When more data become available, the 2 Brazilian groups may be found to be more closely related than illustrated by the tree in Figure 2. No epidemiologic data or clinical manifestations that differentiate isolates from the 2 groups have been reported. Epidemiologic data from recent and current zoonotic outbreaks could help elucidate what the genomic diversity implies. The data do establish a clear connection between group 2 (SAV, BAV, GP1V, and VBH) and NYCBH. VACV-WR is strictly a laboratory strain and has never been used as a vaccine (*29**,**30*). Complete genome sequencing of BRZ-VACV isolates and a sampling of clones from the original seed sample of NYCBH would be a dramatic step toward discerning the relationship between them. What differentiates the 2 lineages—history, ecology or recognizable phenotype—should be further investigated. The circulation of multiple variants across overlapping regions raises the possibility of recombination among variants, which may complicate the evolutionary history of BRZ-VACV.

The certification of global eradication of smallpox was an unprecedented event in human history. However, even now, the origins of VACV in nature and as a vaccine remain a mystery (*3*). During the smallpox eradication campaign, the dogma held that vaccine strains could not survive in nature and that wild-type vaccinia virus was extinct, yet VACV clearly persist today in Brazil and other parts of the world. If these isolates constitute a recently established zoonotic disease, they present a unique opportunity for understanding how molecular changes, such as recombination or other mutations, allow for the adaptation of poxviruses to new hosts and ecologic pressures.

## Supplementary Material

Technical Appendix Nucleotide identity shared among vaccinia strains and isolates for genes E3L, B19R, and A56R*
