# Supplementary material for: Brazilian Vaccinia Viruses and Their Origins
Source: Emerg Infect Dis. 2007 Jul;13(7):965–72. doi: 10.3201/eid1307.061404 (PMC2878226; doi:10.3201/eid1307.061404)
Supplement: Technical Appendix — Nucleotide identity shared among vaccinia strains and isolates for genes E3L, B19R, and A56R* [file 06-1404_Techapp-s1.pdf]

Technical Appendix. Nucleotide identity shared among vaccinia strains and isolates for genes E3L, B19R, and A56R*

E3L

| | RPXV_UTR | VACV_3737 | VACV_Acambis3000 | VACV-MVA | ARAV | VACV_COP | CTGV | GP1V | GP2V | VACV_IOC | VACV-LIS | PSTV | VACV_TianTan | VBH | VACV_WR | HPXV |
|---|---|---|---|---|---|---|---|---|---|---|---|---|---|---|---|---|
| RPXV_UTR | ID | | | | | | | | | | | | | | | |
| VACV_3737 | 0.988 | ID | | | | | | | | | | | | | | |
| VACV_Acambis3000 | 0.983 | 0.986 | ID | | | | | | | | | | | | | |
| VACV-MVA | 0.983 | 0.986 | 1 | ID | | | | | | | | | | | | |
| ARAV | 0.981 | 0.977 | 0.986 | 0.986 | ID | | | | | | | | | | | |
| VACV_COP | 0.979 | 0.986 | 0.992 | 0.992 | 0.99 | ID | | | | | | | | | | |
| CTGV | 0.981 | 0.977 | 0.986 | 0.986 | 1 | 0.99 | ID | | | | | | | | | |
| GP1V | 0.994 | 0.983 | 0.981 | 0.981 | 0.986 | 0.985 | 0.986 | ID | | | | | | | | |
| GP2V | 0.979 | 0.975 | 0.985 | 0.985 | 0.998 | 0.988 | 0.998 | 0.985 | ID | | | | | | | |
| VACV_IOC | 0.994 | 0.983 | 0.988 | 0.988 | 0.986 | 0.985 | 0.986 | 0.992 | 0.985 | ID | | | | | | |
| VACV-LIS | 0.983 | 0.986 | 0.985 | 0.985 | 0.983 | 0.992 | 0.983 | 0.988 | 0.981 | 0.985 | ID | | | | | |
| PSTV | 0.994 | 0.983 | 0.981 | 0.981 | 0.986 | 0.985 | 0.986 | 1 | 0.985 | 0.992 | 0.988 | ID | | | | |
| VACV_TianTan | 0.988 | 0.992 | 0.986 | 0.986 | 0.981 | 0.986 | 0.981 | 0.986 | 0.979 | 0.983 | 0.986 | 0.986 | ID | | | |
| VBH | 0.992 | 0.981 | 0.979 | 0.979 | 0.985 | 0.983 | 0.985 | 0.998 | 0.983 | 0.99 | 0.986 | 0.998 | 0.985 | ID | | |
| VACV_WR | 0.994 | 0.983 | 0.981 | 0.981 | 0.986 | 0.985 | 0.986 | 1 | 0.985 | 0.992 | 0.988 | 1 | 0.986 | 0.998 | ID | |
| HPXV | 0.977 | 0.985 | 0.979 | 0.979 | 0.973 | 0.983 | 0.973 | 0.979 | 0.972 | 0.972 | 0.986 | 0.979 | 0.985 | 0.977 | 0.979 | ID |

B19R

| | **RPXV_UTR** | **VACV_3737** | **VACV_Acambis3000** | **VACV-MVA** | **ARAV** | **VACV_COP** | **CTGV** | **GP1V** | **GP2V** | **VACV_IOC** | **VACV-LIS** | **PSTV** | **VACV_TianTan** | **VBH** | **VACV_WR** | **BFPX_BP1** | **HPXV** |
|---|---|---|---|---|---|---|---|---|---|---|---|---|---|---|---|---|---|
| RPXV_UTR | ID | | | | | | | | | | | | | | | | |
| VACV_3737 | 0.714 | ID | | | | | | | | | | | | | | | |
| VACV_Acambis3000 | 0.974 | 0.693 | ID | | | | | | | | | | | | | | |
| VACV-MVA | 0.974 | 0.693 | 1 | ID | | | | | | | | | | | | | |
| ARAV | 0.992 | 0.715 | 0.973 | 0.973 | ID | | | | | | | | | | | | |
| VACV_COP | 0.99 | 0.71 | 0.966 | 0.966 | 0.993 | ID | | | | | | | | | | | |
| CTGV | 0.992 | 0.715 | 0.973 | 0.973 | 1 | 0.993 | ID | | | | | | | | | | |
| GP1V | 0.99 | 0.712 | 0.968 | 0.968 | 0.993 | 0.991 | 0.993 | ID | | | | | | | | | |
| GP2V | 0.991 | 0.714 | 0.972 | 0.972 | 0.998 | 0.992 | 0.998 | 0.992 | ID | | | | | | | | |
| VACV_IOC | 0.996 | 0.714 | 0.975 | 0.975 | 0.991 | 0.989 | 0.991 | 0.991 | 0.99 | ID | | | | | | | |
| VACV-LIS | 0 | 0 | 0 | 0 | 0 | 0 | 0 | 0 | 0 | 0 | ID | | | | | | |
| PSTV | 0.992 | 0.715 | 0.973 | 0.973 | 1 | 0.993 | 1 | 0.993 | 0.998 | 0.991 | 0 | ID | | | | | |
| VACV_TianTan | 0.991 | 0.714 | 0.969 | 0.969 | 0.996 | 0.994 | 0.996 | 0.992 | 0.995 | 0.99 | 0 | 0.996 | ID | | | | |
| VBH | 0.99 | 0.712 | 0.968 | 0.968 | 0.993 | 0.991 | 0.993 | 1 | 0.992 | 0.991 | 0 | 0.993 | 0.992 | ID | | | |
| VACV_WR | 0.988 | 0.712 | 0.968 | 0.968 | 0.993 | 0.991 | 0.993 | 0.997 | 0.992 | 0.991 | 0 | 0.993 | 0.992 | 0.997 | ID | | |
| BFPX_BP1 | 0.989 | 0.713 | 0.969 | 0.969 | 0.994 | 0.99 | 0.994 | 0.994 | 0.993 | 0.99 | 0 | 0.994 | 0.995 | 0.994 | 0.994 | ID | |
| HPXV | 0.993 | 0.714 | 0.976 | 0.976 | 0.992 | 0.986 | 0.992 | 0.99 | 0.991 | 0.996 | 0 | 0.992 | 0.989 | 0.99 | 0.99 | 0.991 | ID |

A56R

| | **RPXV_UTR** | **VACV_3737** | **VACV_Acambis3000** | **VACV_MVA** | **ARAV** | **BFL-3906** | **BFL-81** | **VACV_COP** | **CTGV** | **GP1V** | **GP2V** | **VACV_IOC** | **VACV_LIS** | **VACV_Malbran** | **PSTV** | **VACV_TianTan** | **VBH** | **VACV_WR** | **HPXV** |
|---|---|---|---|---|---|---|---|---|---|---|---|---|---|---|---|---|---|---|---|
| **RPXV_UTR** | ID | | | | | | | | | | | | | | | | | | |
| **VACV_3737** | 0.957 | ID | | | | | | | | | | | | | | | | | |
| **VACV_Acambis3000** | 0.972 | 0.972 | ID | | | | | | | | | | | | | | | | |
| **VACV_MVA** | 0.972 | 0.972 | 1 | ID | | | | | | | | | | | | | | | |
| **ARAV** | 0.972 | 0.941 | 0.961 | 0.961 | ID | | | | | | | | | | | | | | |
| **BFL-3906** | 0.976 | 0.968 | 0.986 | 0.986 | 0.962 | ID | | | | | | | | | | | | | |
| **BFL-81** | 0.975 | 0.967 | 0.985 | 0.985 | 0.961 | 0.996 | ID | | | | | | | | | | | | |
| **VACV_COP** | 0.98 | 0.976 | 0.989 | 0.989 | 0.958 | 0.987 | 0.986 | ID | | | | | | | | | | | |
| **CTGV** | 0.971 | 0.94 | 0.96 | 0.96 | 0.998 | 0.961 | 0.96 | 0.957 | ID | | | | | | | | | | |
| **GP1V** | 0.971 | 0.969 | 0.989 | 0.989 | 0.962 | 0.985 | 0.984 | 0.981 | 0.961 | ID | | | | | | | | | |
| **GP2V** | 0.964 | 0.933 | 0.955 | 0.955 | 0.991 | 0.954 | 0.953 | 0.95 | 0.993 | 0.956 | ID | | | | | | | | |
| **VACV_IOC** | 0.971 | 0.951 | 0.974 | 0.974 | 0.987 | 0.972 | 0.971 | 0.968 | 0.986 | 0.97 | 0.979 | ID | | | | | | | |
| **VACV_LIS** | 0.971 | 0.974 | 0.994 | 0.994 | 0.962 | 0.987 | 0.986 | 0.99 | 0.961 | 0.988 | 0.954 | 0.97 | ID | | | | | | |
| **VACV_Malbran** | 0.959 | 0.987 | 0.972 | 0.972 | 0.95 | 0.973 | 0.972 | 0.97 | 0.949 | 0.973 | 0.942 | 0.958 | 0.975 | ID | | | | | |
| **PSTV** | 0.971 | 0.94 | 0.96 | 0.96 | 0.998 | 0.961 | 0.96 | 0.957 | 0.997 | 0.961 | 0.99 | 0.986 | 0.961 | 0.949 | ID | | | | |
| **VACV_TianTan** | 0.978 | 0.965 | 0.985 | 0.985 | 0.96 | 0.978 | 0.977 | 0.984 | 0.959 | 0.979 | 0.954 | 0.959 | 0.984 | 0.961 | 0.959 | ID | | | |
| **VBH** | 0.966 | 0.963 | 0.984 | 0.984 | 0.956 | 0.979 | 0.978 | 0.976 | 0.955 | 0.994 | 0.951 | 0.964 | 0.983 | 0.968 | 0.955 | 0.974 | ID | | |
| **VACV_WR** | 0.971 | 0.969 | 0.989 | 0.989 | 0.962 | 0.985 | 0.984 | 0.981 | 0.961 | 1 | 0.956 | 0.97 | 0.988 | 0.973 | 0.961 | 0.979 | 0.994 | ID | |
| **HPXV** | 0.985 | 0.969 | 0.98 | 0.98 | 0.964 | 0.983 | 0.981 | 0.986 | 0.963 | 0.984 | 0.956 | 0.966 | 0.979 | 0.967 | 0.963 | 0.981 | 0.978 | 0.984 | ID |

*RPXV, rabbitpox virus; UTR, Utrecht; VACV, vaccinia virus; MVA, modified vaccinia Ankara; ARAV, Araçatuba virus; COP, Copenhagen; CTGV, Cantagalo virus; GP, Guarani virus; IOC, Oswaldo Cruz Institute; LIS, Lister; PSTV, Passatempo virus; VBH, Belo Horizonte virus; WR, Western Reserve; HPXV, horsepox virus; BFPX, buffalopox virus.
